# The Importance of the C-Terminal Cys Pair of Phosphoribulokinase in Phototrophs in Thioredoxin-Dependent Regulation

**DOI:** 10.1093/pcp/pcac050

**Published:** 2022-04-12

**Authors:** Kazuha Fukui, Keisuke Yoshida, Yuichi Yokochi, Takatoshi Sekiguchi, Ken-ichi Wakabayashi, Toru Hisabori, Shoko Mihara

**Affiliations:** School of Life Science and Technology, Tokyo Institute of Technology, Nagatsuta-cho 4259, Midori-ku, Yokohama 226-8503, Japan; School of Life Science and Technology, Tokyo Institute of Technology, Nagatsuta-cho 4259, Midori-ku, Yokohama 226-8503, Japan; Laboratory for Chemistry and Life Science, Institute of Innovative Research, Tokyo Institute of Technology, Nagatsuta-cho 4259, Midori-ku, Yokohama 226-8503, Japan; School of Life Science and Technology, Tokyo Institute of Technology, Nagatsuta-cho 4259, Midori-ku, Yokohama 226-8503, Japan; School of Life Science and Technology, Tokyo Institute of Technology, Nagatsuta-cho 4259, Midori-ku, Yokohama 226-8503, Japan; School of Life Science and Technology, Tokyo Institute of Technology, Nagatsuta-cho 4259, Midori-ku, Yokohama 226-8503, Japan; Laboratory for Chemistry and Life Science, Institute of Innovative Research, Tokyo Institute of Technology, Nagatsuta-cho 4259, Midori-ku, Yokohama 226-8503, Japan; School of Life Science and Technology, Tokyo Institute of Technology, Nagatsuta-cho 4259, Midori-ku, Yokohama 226-8503, Japan; Laboratory for Chemistry and Life Science, Institute of Innovative Research, Tokyo Institute of Technology, Nagatsuta-cho 4259, Midori-ku, Yokohama 226-8503, Japan; Laboratory for Chemistry and Life Science, Institute of Innovative Research, Tokyo Institute of Technology, Nagatsuta-cho 4259, Midori-ku, Yokohama 226-8503, Japan

**Keywords:** Calvin–Benson cycle, Cyanobacteria, Phosphoribulokinase, Redox regulation, Thioredoxin

## Abstract

Phosphoribulokinase (PRK), one of the enzymes in the Calvin–Benson cycle, is a well-known target of thioredoxin (Trx), which regulates various enzyme activities by the reduction of disulfide bonds in a light-dependent manner. PRK has two Cys pairs conserved in the N-terminal and C-terminal regions, and the N-terminal one near the active site is thought to be responsible for the regulation. The flexible clamp loop located between the N-terminal two Cys residues has been deemed significant to Trx-mediated regulation. However, cyanobacterial PRK is also subject to Trx-dependent activation despite the lack of this clamp loop. We, therefore, compared Trx-mediated regulation of PRK from the cyanobacterium *Anabaena* sp. PCC 7120 (A.7120_PRK) and that from the land plant *Arabidopsis thaliana* (AtPRK). Interestingly, peptide mapping and site-directed mutagenesis analysis showed that Trx was more effective in changing the redox states of the C-terminal Cys pair in both A.7120_PRK and AtPRK. In addition, the effect of redox state change of the C-terminal Cys pair on PRK activity was different between A.7120_PRK and AtPRK. Trx-mediated redox regulation of the C-terminal Cys pair was also important for complex dissociation/formation with CP12 and glyceraldehyde 3-phosphate dehydrogenase. Furthermore, in vivo analysis of the redox states of PRK showed that only one disulfide bond is reduced in response to light. Based on the enzyme activity assay and the complex formation analysis, we concluded that Trx-mediated regulation of the C-terminal Cys pair of PRK is important for activity regulation in cyanobacteria and complex dissociation/formation in both organisms.

## Introduction

The photosynthetic carbon fixation cycle, Calvin–Benson cycle, is highly regulated in response to environmental changes. Most photosynthetic organisms have a thiol-based redox regulation system that allows for the light-dependent activation of enzymes in the Calvin–Benson cycle. In this system, thioredoxin (Trx) transfers the reducing equivalents from the photosynthetic electron transport chain to their target proteins by dithiol-disulfide exchange reactions (Buchanan 1980, Buchanan and Balmer 2005).

One of the Calvin–Benson cycle enzyme phosphoribulokinase (PRK) catalyzes the adenosine triphosphate (ATP)-dependent phosphorylation of ribulose 5-phosphate (Ru5P) to ribulose 1,5-bisphosphate. This PRK is known as a redox-regulated enzyme governed by the chloroplast Trx (Wolosiuk and Buchanan 1978). Based on the amino acid sequence homology, PRK can be classified into four groups: class IA, found in land plants, green algae, β-cyanobacteria and red algae; class IB, found in chromista, dinoflagellates and haptophytes; class II found in archaea and; class III found in proteobacteria and α-cyanobacteria (Wilson et al. 2019). Class IA PRK is deactivated by the redox-dependent formation of a complex with the inhibitor protein CP12 and another Calvin–Benson cycle enzyme glyceraldehyde 3-phosphate dehydrogenase (GAPDH), or the formation of an intramolecular disulfide bond (Hirasawa et al. 1998, Marri et al. 2005). Two conserved Cys pairs on the PRK molecule are reported to be involved in this thiol-based regulation: one close to the ATP-binding site (N-terminal Cys pair) and the other close to the interface of the PRK homodimer (C-terminal Cys pair; Gurrieri et al. 2019, McFarlane et al. 2019, Wilson et al. 2019, Yu et al. 2020; **Supplementary Fig. S1**). It is already reported that the redox states of the N-terminal and C-terminal Cys pairs affect the enzyme activity and complex formation with CP12 and GAPDH, respectively (Hirasawa et al. 1998, Thieulin-Pardo et al. 2015, Yu et al. 2020).

There are many reports on the biochemical analyses of the redox-dependent activation and inactivation of the enzyme activity of PRK so far (Wolosiuk and Buchanan 1978, Hirasawa et al. 1998, Geck and Hartman 2000, Marri et al. 2009). Trx was thought to interact with the N-terminal Cys pair and regulates PRK activity in a light-dependent manner (Brandes et al. 1996). Previous study has suggested that the flexible clamp loop located at the N-terminus contributes to disulfide bond formation between the N-terminal Cys residues based on the structural analyses (Gurrieri et al. 2019; **Supplementary Fig. S1**). Interestingly, a recent study has suggested that Trx enhances the activity of PRK of the cyanobacterium *Microcystis aeruginosa* PCC 7806, which lacks the clamp loop region (Hackenberg et al. 2018).

In this study, we, therefore, investigated Trx-mediated redox regulation of PRK from the cyanobacteria *Anabaena* sp. PCC 7120 (*A.*7120) and the land plant *Arabidopsis thaliana* to elucidate how PRK is redox-regulated in phototrophs at the molecular level. Contrary to previous reports, our in vitro analyses demonstrated that Trx is effective in changing the redox states of the C-terminal Cys pair of PRK from both cyanobacteria and plants and contributes to the redox regulation of this enzyme.

## Results

### Trx alters redox state of the C-terminal Cys pair of A.7120_PRK

**Fig. 1:**
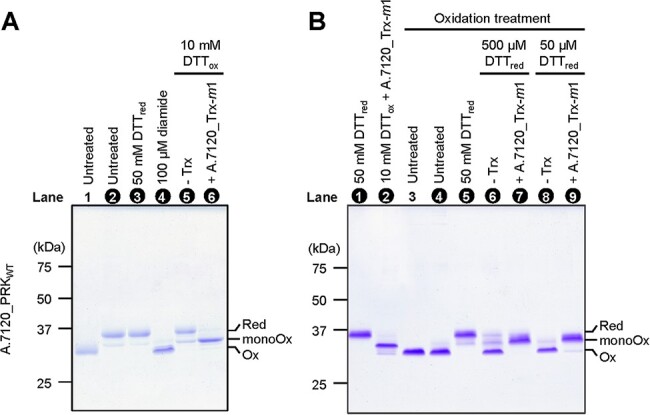
Redox responses of cyanobacterial PRK in vitro. (A) A.7120_PRK_WT_ (2 µM) was incubated with 50 mM DTT_red_, 100 µM diamide or 1 µM A.7120_Trx-*m*1 in the presence of 10 mM DTT_ox_. Proteins were then precipitated with 10% (w/v) TCA, labeled with SDS sample buffer containing 2 mM PEO, subjected to nonreducing SDS-PAGE and stained with CBB. (B) A.7120_PRK_WT_ (40 µM) was treated with 100 µM diamide and desalted. A.7120_PRK_WT_ (2 µM) was then incubated with 50 or 500 µM DTT_red_, and 1 µM A.7120_Trx-*m*1. A.7120_PRK_WT_ without pretreatment was incubated with 50 mM DTT_red_ or with 1 µM A.7120_Trx-*m*1 in the presence of 10 mM DTT_ox_, and subjected as control (lanes 1 and 2). (A and B) The redox states of A.7120_PRK were determined as described using the PEO labeling method (A: lanes 2–6 and B: lanes 1, 2, 4–9), and those lane numbers were indicated by white letters. Presumed redox states of A.7120_PRK are indicated as Red, reduced form; monoOx, mono-oxidized form; Ox, oxidized form.

We prepared a recombinant PRK of *A*.7120 (A.7120_PRK) and examined the redox response of this enzyme in the presence or absence of recombinant Trx-*m*1 (A.7120_Trx-*m*1), a major Trx isoform in *A*.7120, using the thiol-modifying reagent maleimide-PEG_11_-biotin (PEO). Consequently, three redox states of A.7120_PRK were observed (**Fig. 1**). Because A.7120_PRK possesses two Cys pairs in the molecule, these three forms must correspond to the enzyme molecule containing 0, 1 and 2 intramolecular disulfide bonds, respectively. We, therefore, designated these three bands as the reduced form (Red), the mono-oxidized form (monoOx) and the fully oxidized form (Ox), respectively. A.7120_PRK was purified as a mixture of the mono-oxidized form and the reduced form (**Fig. 1A**, lane 2), and the fully oxidized form was detected when incubated with 100 µM diamide for 30 min (**Fig. 1A**, lane 4). Treatment with 10 mM oxidized dithiothreitol (DTT_ox_) did not primarily affect the initial redox states (**Fig. 1A**, lane 5). After incubation with 1 µM A.7120_Trx-*m*1 and 10 mM DTT_ox_, the redox state shifted from the reduced form to the mono-oxidized form (**Fig. 1A**, lane 6). These results indicate that A.7120_Trx-*m*1 can oxidize only one Cys pair on the PRK molecule under this experimental condition.

 Contrary to these experiments, we examined the reduction process in the following way. At first, A.7120_PRK was pretreated with 100 µM diamide for oxidation. When the initially oxidized A.7120_PRK (**Fig. 1B**, lane 4) was incubated with 500 or 50 µM reduced dithiothreitol (DTT_red_), the protein was not reduced very much (**Fig. 1B**, lanes 6 and 8). After incubation with 1 µM A.7120_Trx-*m*1 in the presence of DTT_red_, the redox state of A.7120_PRK has shifted to the mono-oxidized form (**Fig. 1B**, lanes 7 and 9). These results clearly indicate that A.7120_Trx-*m*1 reduced only one Cys pair on the PRK molecule.

**Fig. 2:**
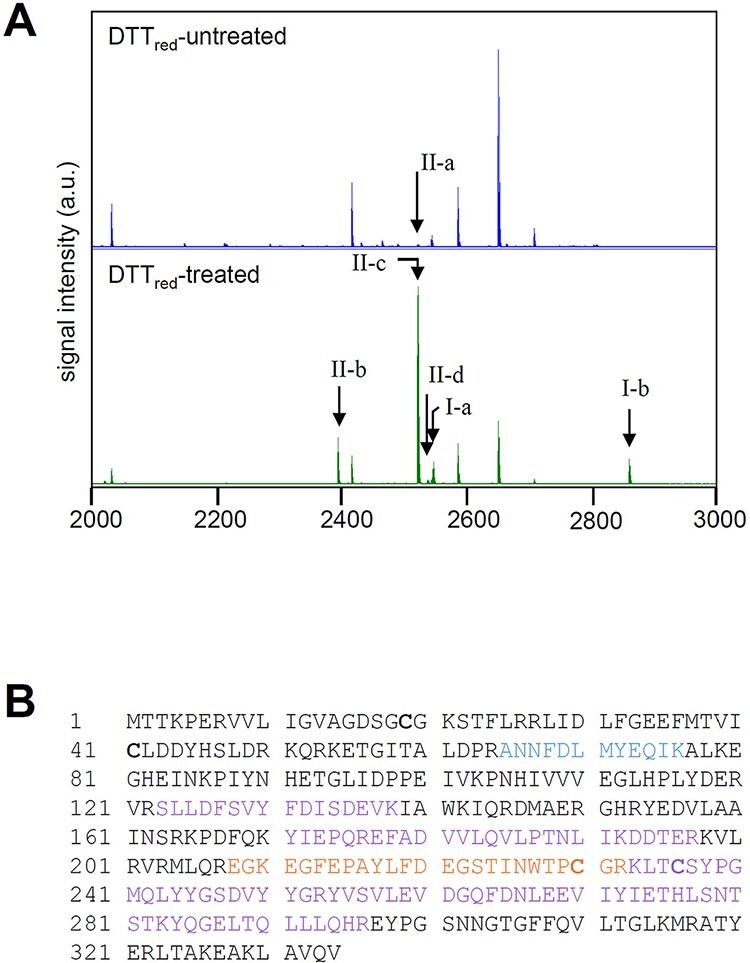
Identification of redox-active Cys residues of cyanobacterial PRK. (A) MALDI-TOF MS spectra of A.7120_PRK. After incubation of A.7120_PRK with DTT_ox_ and A.7120_Trx-*m*1, PRK was precipitated with 10% (w/v) TCA, labeled with SDS sample buffer containing 2 mM PEO, subjected to nonreducing SDS-PAGE and stained with CBB. Protein band of the mono-oxidized form of PRK was excised from the gel and divided into two pieces. One piece was treated with 10 mM DTT_red_ and then 55 mM IAA for alkylation (DTT_red_ treated). The other was treated with 55 mM IAA but not with DTT_red_ (DTT_red_-untreated). After proteins were in-gel digested with trypsin, each sample was analyzed by MALDI-TOF MS. Arrows indicate a specific peak matched to the peptide, including C230 (I-a and I-b) or C236 (II-a, II-b, II-c and II-d) alkylated by IAA. (B) The matched peptides using the search engine. Peptide fractions detected only in the DTT_red_-treated sample, only in DTT_red_-untreated sample, and in both samples are indicated in red, blue and purple, respectively.

 As described above, A.7120_PRK possesses two Cys pairs: the N-terminal (Cys19 and Cys41) and the C-terminal pairs (Cys230 and Cys236). We performed peptide mapping analysis to determine the Cys pair involving A.7120_Trx-*m*1. After the oxidation of A.7120_PRK with A.7120_Trx-*m*1 and DTT_ox_, the mono-oxidized form was separated by PEO labeling followed by nonreducing sodium dodecyl sulfate-polyacrylamide gel electrophorsis (SDS-PAGE) as described above. The isolated protein band was divided into two pieces. Only one piece was treated with DTT_red_ to reduce the disulfide bond of the redox-responsive Cys pair of PRK before treated with iodoacetamide (IAA). Then, both pieces were incubated with trypsin for in-gel digestion. The resulting peptides were analyzed by matrix-assisted laser desorption/ionization-time of flight mass spectrometry (MALDI-TOF MS) (see figure legends for details). The redox-responsive Cys pair was alkylated only in the DTT_red_-treated sample. The sequence recovery of the MALDI-TOF MS analysis of A.7120_PRK untreated or treated with DTT_red_ was 35 and 39%, respectively. The specific peaks at *m*/*z* 2546.1940 (**Fig. 2A**, I-a) and 2860.3470 (**Fig. 2A**, I-b) were observed only in the peptides obtained from the DTT_red_-treated sample (**Fig. 2A** and **Table 1**). These values correspond to the calculated mass of tryptic peptides containing Cys230 alkylated with IAA (**Fig. 2B**, orange-colored letters and **Table 1**). Furthermore, three peaks at *m*/*z* 2393.1060 (**Fig. 2A**, II-b), 2521.2000 (**Fig. 2A**, II-c) and 2537.1890 (**Fig. 2A**, II-d) were observed predominantly in the DTT_red_-treated sample, although a faint peak at *m*/*z* 2521.1860 (**Fig. 2A**, II-a) was observed in the untreated sample (**Fig. 2A** and **Table 1**). The mass values of these peaks correspond to the calculated mass of the tryptic peptide containing Cys236 alkylated with IAA (**Fig. 2B** and **Table 1**). These results suggest that the C-terminal Cys pair in A.7120_PRK is redox-regulated by A.7120_Trx-*m*1.

**Table 1:** Peptide mapping analysis of A.7120_PRK

Peptide mass		
Observed^a^	Calculated^b^	Peptide fragment^c^
**DTT_red_-untreated**		
1485.7230	1484.6969	^65^ANNFDLMYEQIK^76^
1876.9460	1875.9142	^123^SLLDFSVYFDISDEVK^138^
2585.4770	2584.4265	^171^YIEPQREFADVVLQVLPTNLIK^192^
2415.3100	2414.2693	^177^EFADVVLQVLPTNLIKDDTER^197^
2521.1860	2520.1454	^233^KLTCSYPGMQLYYGSDVYYGR^253^ (II-a in **Fig. 2A**)
3442.7330	3441.6828	^254^YVSVLEVDGQFDNLEEVIYIETHLSNTSTK^283^
1598.8850	1597.8576	^284^YQGELTQLLLQHR^296^
**DTT_red_-treated**		
1876.9870	1875.9142	^123^SLLDFSVYFDISDEVK^138^
2585.5130	2584.4265	^171^YIEPQREFADVVLQVLPTNLIK^192^
1799.0840	1798.0240	^177^EFADVVLQVLPTNLIK^192^
2415.3440	2414.2693	^177^EFADVVLQVLPTNLIKDDTER^197^
2860.3470	2859.2810	^208^ **EGKEGFEPAYLFDEGSTINWTPCGR** ^232^ (I-b in **Fig. 2A**)
2546.1940	2545.1220	^211^ **EGFEPAYLFDEGSTINWTPCGR** ^232^ (I-a in **Fig. 2A**)
2521.2000	2520.1454	^233^KLTCSYPGMQLYYGSDVYYGR^253^ (II-c in **Fig. 2A**)
2537.1890	2536.1403	^233^KLTCSYPGMQLYYGSDVYYGR^253^ + Oxidation (M) (II-d in **Fig. 2A**)
2393.1060	2392.0504	^234^ **LTCSYPGMQLYYGSDVYYGR** ^253^ (II-b in **Fig. 2A**)
3442.7880	3441.6828	^254^YVSVLEVDGQFDNLEEVIYIETHLSNTSTK^283^
1598.9080	1597.8576	^284^YQGELTQLLLQHR^296^

a Molecular mass of peptide in the experiment.

b Molecular mass of peptide in calculation. Every Cys was calculated as carbamidomethyl Cys.

c Numbers shown on the sequences correspond to amino acid positions in A.7120_PRK. The peptides obtained from only the DTT_red_-treated sample are indicated in bold.

**Fig. 3:**
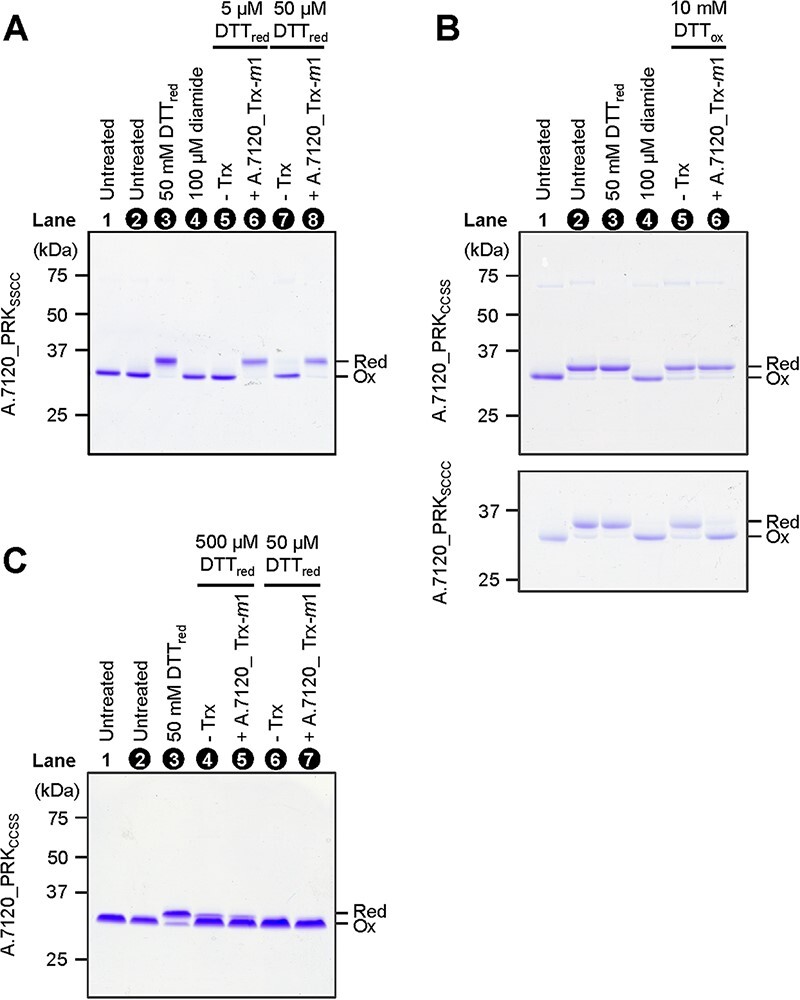
Redox state changes in Cys mutants of A.7120_PRK in vitro. (A) and (B) PRK_SSCC_ (2 µM), PRK_CCSS_ (2 µM) or PRK_SCCC_ (2 µM) was incubated with 50 mM DTT_red_, 100 µM diamide, 1 µM A.7120_Trx-*m*1 and 5–50 µM of DTT_red_, or 1 µM A.7120_Trx-*m*1 and 10 mM DTT_ox_. Proteins were then precipitated with 10% (w/v) TCA and stained with CBB. (C) A.7120_PRK_CCSS_ (40 µM) was treated with 100 µM diamide and desalted. A.7120_PRK_CCSS_ (2 µM) was incubated with 50 mM DTT_red_ or 50–500 µM DTT_red_ and 1 µM A.7120_Trx-*m*1. (A, B, C) The redox states of Cys mutants of A.7120_PRK were determined by PEO labeling as described, and those lane numbers were indicated by white letters. A.7120_PRK unlabeled by PEO was subjected to lane 1. Presumed redox states of A.7120_PRK are indicated on the right. Red, reduced form; Ox, oxidized form.

 To further investigate the target Cys pair of A.7120_Trx-*m*1, we prepared the mutant enzymes whose N-terminal or C-terminal Cys residues were substituted with Ser, and these proteins were designated as A.7120_PRK_SSCC_ and A.7120_PRK_CCSS_, respectively. In addition, we prepared the single Cys mutant of the N-terminal Cys pair (A.7120_PRK_SCCC_). A.7120_PRK_SSCC_ was purified as the oxidized form and reduced by A.7120_Trx-*m*1 in the presence of a low concentration of DTT_red_ (**Fig. 3A**, lanes 2, 6 and 8). In contrast, A.7120_PRK_SCCC_ and A.7120_PRK_CCSS_ were purified as the reduced form (**Fig. 3B**, lane 2). A.7120_PRK_SCCC_ was then oxidized by A.7120_Trx-*m*1 in the presence of DTT_ox_, whereas A.7120_PRK_CCSS_ was not (**Fig. 3B**, lane 6). In the case of A.7120_PRK_CCSS_, which was pretreated with diamide for oxidation (**Fig. 3C**, lane 2), A.7120_Trx-*m*1 could not reduce the protein at all even in the presence of 50 or 500 µM DTT_red_ (**Fig. 3C**, lanes 5 and 7), suggesting again that the C-terminal Cys pair is the target of A.7120_Trx-*m*1 for reduction.

### A.7120_PRK activity is partially inhibited by oxidation of the C-terminal Cys pair

**Fig. 4:**
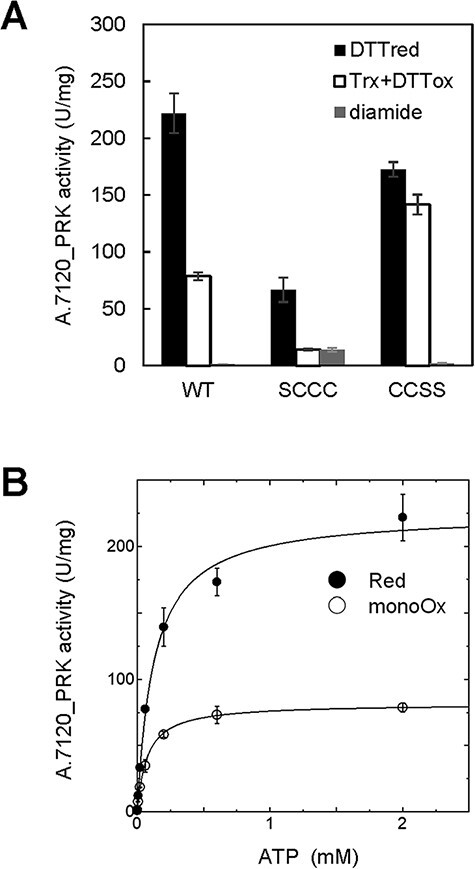
Redox sensitivity of cyanobacterial PRK and its cysteine mutants. (A) After A.7120_PRK_WT_, A.7120_PRK_SCCC_ or A.7120_PRK_CCSS_ was incubated with 50 mM DTT_red_ (black bar), 100 µM diamide (gray bar) or 1 µM A.7120_Trx-*m*1 and 10 mM DTT_ox_ (white bar) at 30°C for 30 min, PRK activity was measured under 2 mM ATP conditions. A.7120_PRK_SSCC_ did not show any activity at all. Data are represented as mean ± SD (*n* = 3–6). (B) The activity of the reduced form and the mono-oxidized form of A.7120_PRK_WT_ were measured under varying ATP concentrations. Data are represented as mean ± SD (*n* = 3–6) and fitted to the Michaelis–Menten equation.

We then investigated the relationship between A.7120_PRK activity and its redox state in vitro. A.7120_PRK was incubated with oxidants, reductants or A.7120_Trx-*m*1 under the same conditions as **Fig. 1**, and its activity was measured. When A.7120_PRK was incubated with diamide in advance for oxidation, the oxidized form of A.7120_PRK was inactive (**Fig. 4A**, gray bar). Compared to the reduced form of A.7120_PRK (**Fig. 4A**, black bar), the mono-oxidized form of A.7120_PRK showed lower activity (**Fig. 4A**, white bar). We also examined the PRK activity of the Cys mutants. A.7120_PRK_SSCC_ did not show any activity at all. Although the activities of A.7120_PRK_SCCC_ were lower than those of wild-type, A.7120_PRK_SCCC_ was also deactivated by A.7120_Trx-*m*1 in the presence of DTT_ox_ (**Fig. 4A**, white bar). Unlike wild-type enzyme and A.7120_PRK_SCCC_, the oxidation treatment with A.7120_Trx-*m*1 and DTT_ox_ did not significantly suppress A.7120_PRK_CCSS_ activity (**Fig. 4A**, white bar) whereas the oxidized form did not show any activity (**Fig. 4A**, gray bar). These results imply that Trx regulates A.7120_PRK activity by changing the redox states of the C-terminal Cys pair. In contrast to A.7120_PRK_WT_ and A.7120_PRK_CCSS_, A.7120_PRK_SCCC_ retained the residual activity irrespective of the oxidation conditions (**Fig. 4A**, gray bar), implying that oxidation of the N-terminal Cys pair of A.7120_PRK results in a complete loss of activity.

 To investigate the difference between the reduced form and the mono-oxidized form of A.7120_PRK, we measured the activity under various ATP concentrations. A.7120_PRK showed Michaelis–Menten-type kinetics (**Fig. 4B**). The kinetic parameters were determined by fitting the data to the Michaelis–Menten equation (**Table 2**). The *V*_max_ value was significantly decreased in the mono-oxidized form (*P* = 0.00083, Welch’s *t*-test). The *K*_m_ value of the mono-oxidized form was slightly lower than that of the reduced form (*P* = 0.096, Welch’s *t*-test).

**Table 2:** Kinetic parameters of A.7120_PRK and AtPRK. PRK activities were measured under varying ATP concentrations. *V*_max_ and *K*_m_ for ATP were estimated by fitting the data to the Michaelis–Menten equation. These values are represented as mean ± SD (*n* = 3)

Species	Redox states	*V*_max_ (U/mg)	*K*_m_ (µM)
A.7120_PRK	Reduced form	214 ± 11.0	109 ± 4.5
A.7120_PRK	Mono-oxidized form	81.4 ± 5.4	76.7 ± 16.4
AtPRK	Reduced form	289 ± 34.0	46.5 ± 6.5
AtPRK	Mono-oxidized form	262 ± 15.0	64.0 ± 20.0

### Only one Cys pair is reduced in a light-dependent manner in cyanobacteria

**Fig. 5:**
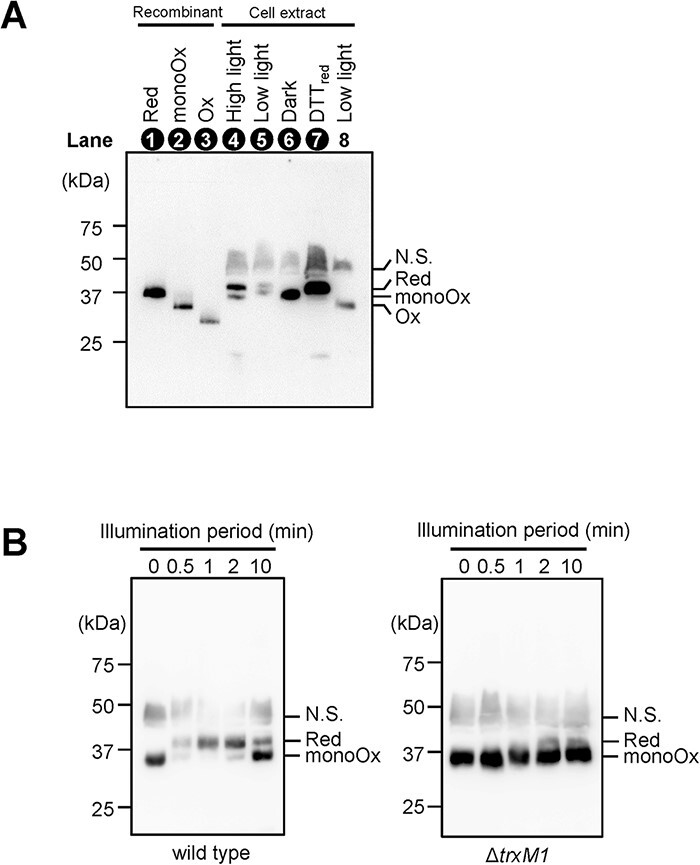
Redox states of PRK in *A.*7120 cells. (A) Redox states of PRK under high-light (200 µmol photons m^−2^ s^−1^), low-light (30 µmol photons m^−2^ s^−1^), dark (dark-adapted for 3 h) and DTT-treated (DTT: treated with 25 mM DTT_red_) conditions were determined by immunoblot using anti-AtPRK antibodies. The redox states of A.7120_PRK were determined as described using the PEO labeling method (lanes 1–7), and those lane numbers were indicated by white letters. Red, reduced form; monoOx, mono-oxidized form; Ox, oxidized form. N.S. indicates nonspecific bands. Recombinant A.7120_PRK (8 ng) was subjected as control (lanes 1–3). (B) The time courses of PRK reduction in *A*.7120 and *trxM1* knockout mutant were indicated. After dark adaptation for 3 h, the cells were illuminated under low-light conditions (30 µmol photons m^−2^ s^−1^) for the indicated period, and the redox states of PRK were determined.

We investigated the in vivo redox state of the protein using PEO labeling and immunoblot analysis to reveal the change in redox states of A.7120_PRK under physiological conditions. The apparent molecular mass of the mature protein in cyanobacteria was observed to be larger than that of the recombinant A.7120_PRK_WT_ (**Fig. 5A**, lanes 1 and 7; both are the reduced forms). In order to distinguish proteins by the chemical modification of Cys using 4-acetamido-4ʹ-maleimidylstilbene-2,2ʹ-disulfonate (AMS) or PEO, the proteins in the gel must be separated without pretreatment by reducing the sample with 2-mercaptoethanol. Because cell extracts contain a variety of proteins, this will affect the mobility of each protein in the gel under this experimental condition, resulting in an apparent difference in molecular size between the protein of interest in the cell extract and the recombinant. Since A.7120_PRK has four cys residues, which can form two disulfide bonds under the oxidizing conditions, the migration of the protein that is completely oxidized and then PEO labeled should be the same as the protein without labeling (**Fig. 1A**, lane 1 and 4). However, the apparent protein size of PRK in the cell without PEO labeling (**Fig. 5A**, lane 8) was again larger than the fully oxidized recombinant protein (**Fig. 5A**, lane 3). If this is the case, the apparent molecular weight of PRK in vivo is larger than that of the recombinant protein, and the protein shown in lane 6 of **Fig. 5A** probably contains only one disulfide bond (labeled as ‘monoOx’), since its molecular weight is larger than that shown in lane 8. In contrast, a part of A.7120_PRK was detected as the reduced form in the light (**Fig. 5A**, lanes 4 and 5). The ratio of the reduced form to the mono-oxidized form increased with the intensity of the light (**Fig. 5A**, lanes 4 and 5). These results imply that one Cys pair of A.7120_PRK does not form a disulfide bond in vivo even in the dark, whereas the other shows a light intensity-dependent redox change. This reduction of the disulfide bond was suppressed in the *trxM1* (alr0052: the gene for Trx-*m*1) deficient mutant strain (*∆trxM1*) (**Fig. 5B**), indicating Trx-*m*1 is the major reductant of A.7120_PRK in cells.

### C-terminal Cys pair of plant-type PRK is reduced/oxidized by Trx

Plant-type PRK possesses a flexible clamp loop at the N-terminus. We hypothesized that, because the amino acid sequence around the C-terminal Cys pair is similar to that of A.7120_PRK (**Supplementary Fig. S2**), Trx regulates the redox state of the C-terminal Cys pair of plant-type PRK in addition to the N-terminal Cys pair, whose redox sensitivity has already been reported (Thieulin-Pardo et al. 2015). We examined the effect of Trx on the redox state of PRK from *A. thaliana* (AtPRK) using AMS. Arabidopsis Trx-*f*1 (AtTrx-*f*1) and Trx-*m*2 (AtTrx-*m*2) were used in this study because these Trx isoforms have already been reported to activate AtPRK (Marri et al. 2009).

**Fig. 6:**
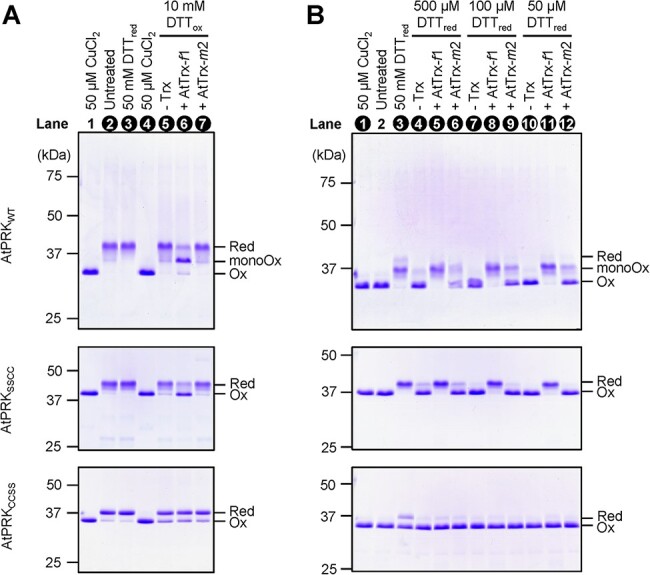
Redox responses of plant PRK and its cysteine mutants in vitro. (A) AtPRK_WT_ (2 µM), AtPRK_SSCC_ (2 µM) or AtPRK_CCSS_ (2 µM) was incubated with 50 mM DTT_red_, 50 µM CuCl_2_, or 10 mM DTT_ox_ and 1 µM Trx. Proteins were then precipitated with 10% (w/v) TCA, labeled with SDS sample buffer containing 2 mM AMS, subjected to nonreducing SDS-PAGE and stained with CBB. The redox states of AtPRK were determined by AMS labeling as described (lanes 2–7), and those lane numbers were indicated by white letters. (B) AtPRK (25 µM) was treated with 50 µM CuCl_2_ and desalted. AtPRK_WT_ (2 µM), AtPRK_SSCC_ (2 µM) or AtPRK_CCSS_ (2 µM) was incubated with 50 mM DTT_red_ or 50–500 µM DTT_red_ and 1 µM Trx. The redox states of AtPRK were determined by AMS labeling as described (lanes 1 and 3–12), and those lane numbers were indicated by white letters. Presumed redox states of AtPRK are indicated on the right. Red, reduced form; monoOx, mono-oxidized form; Ox, oxidized form.

Purified recombinant AtPRK_WT_ was mostly present as the reduced form and entirely oxidized by the oxidant CuCl_2_ (**Fig. 6A**, lanes 2 and 4). Interestingly, when incubated with AtTrx-*f*1 and DTT_ox_, AtPRK_WT_ was mostly present as the mono-oxidized form but not as the fully oxidized form (**Fig. 6A**, lane 6). Furthermore, AtTrx-*m*2 did not affect the redox state of AtPRK_WT_ under the studied condition (**Fig. 6A**, lane 7). We also investigated the redox responses of the Cys mutants, N-terminal Cys mutant (AtPRK_SSCC_) and C-terminal Cys mutant (AtPRK_CCSS_), to determine the redox-regulated Cys pair in AtPRK. Purified AtPRK_SSCC_ and AtPRK_CCSS_ were mostly present as the reduced form (**Fig. 6A**, lane 2). AtPRK_SSCC_ was oxidized by AtTrx-*f*1 in the presence of DTT_ox_, as was AtPRK_WT_ but not by AtTrx-*m*2 (**Fig. 6A**, lanes 6 and 7). AtPRK_CCSS_ was partially oxidized by DTT_ox_ even without Trxs (**Fig. 6A**, lane 5), and neither AtTrx-*f*1 nor AtTrx-*m*2 accelerated this oxidation (**Fig. 6A**, lanes 6 and 7). Then, the reduction process was examined. AtPRK_WT_, AtPRK_SSCC_ and AtPRK_CCSS_ were completely oxidized by pretreatment with CuCl_2_ (**Fig. 6B**, lanes 1 and 2). The oxidized AtPRK_WT_ and AtPRK_CCSS_ were not fully reduced even in the presence of 50 mM DTT_red_ (**Fig. 6B**, lane 3). When incubated with DTT_red_ and AtTrx-*f*1 or AtTrx-*m*2, the redox state of AtPRK_WT_ was shifted to mono-oxidized form (**Fig. 6B**, lanes 5, 6, 8, 9, 11 and 12). In addition, AtPRK_SSCC_, but not AtPRK_CCSS_, was fully reduced by Trx-*f*1 in the presence of DTT_red_ (**Fig. 6B**, lanes 5, 8 and 11). Taken together, these results indicate that the C-terminal Cys pair of AtPRK is also the target of Trx for reduction/oxidation.

**Fig. 7:**
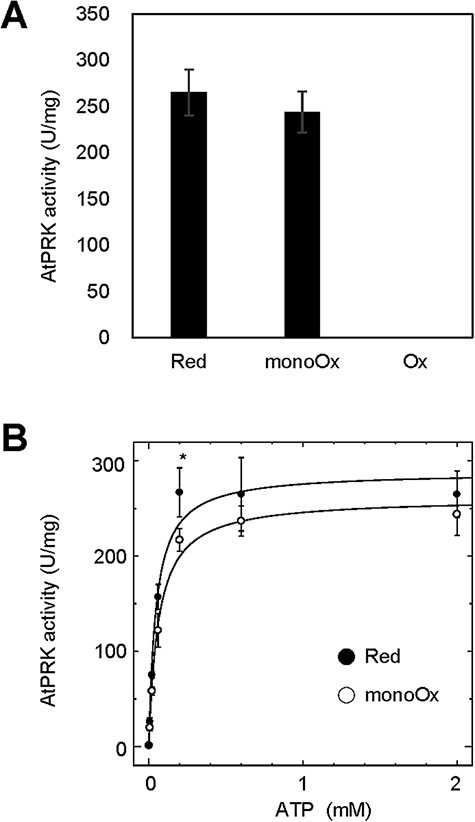
Effects of redox states on plant PRK activity. (A) After 2 µM AtPRK_WT_ was treated with 50 mM DTT_red_, 50 µM CuCl_2_, or 1 µM AtTrx-*f*1 and 10 mM DTT_ox_, PRK activities were measured under 2 mM ATP conditions. Data are represented as mean ± SD (*n* = 3). (B) The activities of the reduced form and the mono-oxidized form of AtPRK_WT_ were measured under varying ATP concentrations (ranging from 0 to 2 mM). Data are represented as mean ± SD (*n* = 3) and fitted using the Michaelis–Menten equation. An asterisk indicates a statistically significant difference in activity between the reduced form and the mono-oxidized form (Student’s *t*-test, *P* < 0.05).

We then examined whether the activity of AtPRK_WT_ is redox-regulated by AtTrx-*f*1. PRK activities were measured after treatment with DTT_red_, CuCl_2_, or AtTrx-*f*1 and DTT_ox_. The oxidized form of AtPRK_WT_ was inactive (**Fig. 7A**). In contrast to A.7120_PRK, the activity of the mono-oxidized enzyme form AtPRK_WT_ was almost the same as that of the reduced one (**Fig. 7A**). We also measured PRK activity under various ATP concentrations. Both the reduced form and the mono-oxidized form enzymes showed Michaelis–Menten-type kinetics (**Fig. 7B**). The kinetic parameters were then determined by fitting the data to the Michaelis–Menten equation (**Table 2**). Consequently, there were no significant differences in activity, *K*_m_ values for ATP, or *V*_max_ values between the reduced form and the mono-oxidized form except for the activities at 200 µM ATP.

**Fig. 8:**
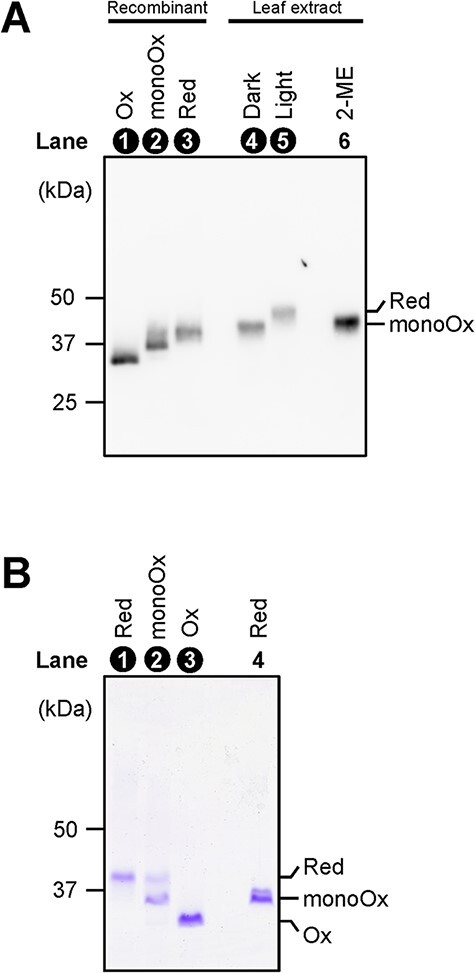
Redox states of PRK in *Arabidopsis* leaves. (A) Redox states of PRK under dark and light conditions were determined as described in the Materials and Methods section. The apparent molecular mass of PRK detected in the AMS-labeled dark-adapted sample (lane 4), AMS-labeled irradiated sample (lane 5) and unlabeled leaf extract treated with 2-mercaptoethanol (2-ME) (lane 6) were compared. Recombinant AtPRK was subjected as control (lanes 1–3). (B) The apparent molecular mass of PRKs was compared among the AMS-labeled reduced form (lane 1), mono-oxidized form (lane 2), oxidized form (lane 3) and unlabeled reduced form (lane 4). In the lanes numbered in white, AMS-labeled samples were analyzed.

Finally, we examined the in vivo redox states of AtPRK. In *Arabidopsis* leaves, AtPRK was reduced by illumination (**Fig. 8A**, lanes 4 and 5). We used recombinant AtPRK_WT_ as a control, but the apparent molecular mass of the mature protein in chloroplasts was larger than that of recombinant protein (**Fig. 8A**). We therefore simply evaluated the band shifts of the western blotting signals resulting from the change in redox state and compared them with the changes in mobility of bands of the recombinant proteins on the Coomassie Brilliant Blue (CBB)-stained SDS-PAGE gel. The apparent molecular mass of the AMS-labeled reduced form was larger, while that of the oxidized form was smaller than that of the unlabeled reduced form (**Fig. 8B**, lanes 1, 3 and 4). The apparent molecular mass of the mono-oxidized form was the same as that of the unlabeled reduced form (**Fig. 8B**, lanes 2 and 4). The apparent molecular mass of PRK detected in the AMS-labeled dark-adapted sample was the same as that in the unlabeled leaf extract treated with 2-mercaptoethanol (**Fig. 8A**, lanes 4 and 6) whereas PRK must be present as the reduced form in the light (**Fig. 8A**, lane 5). Because the apparent molecular mass of PRK detected in the AMS-labeled illuminated sample was observed to be larger than that in the unlabeled leaf extract treated with 2-mercaptoethanol (**Fig. 8A**, lanes 5 and 6), we suspected that AtPRK was present as the mono-oxidized and reduced forms under dark and light conditions, respectively.

### Trx-mediated redox regulation of AtPRK is important for complex formation/dissociation

**Fig. 9:**
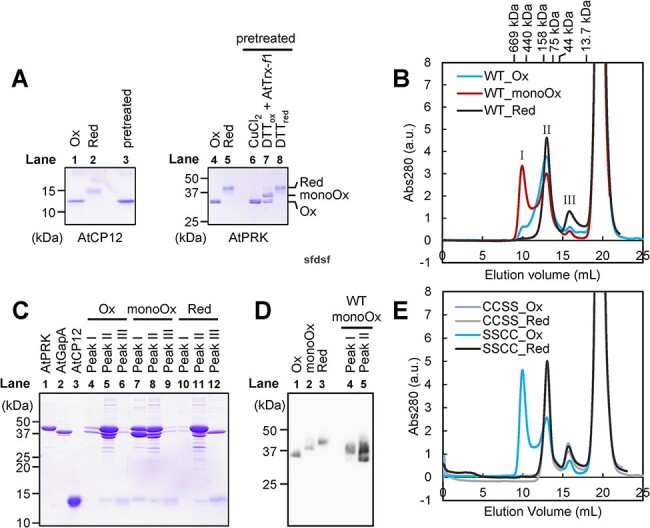
Complex formation ability of AtPRK. (A) Redox states of AtCP12 treated with CuCl_2_ and AtPRK treated with CuCl_2_, DTT_red_, or DTT_ox_ and AtTrx-*f*1 were examined by nonreducing SDS-PAGE. AtCP12 and AtPRK incubated with 50 µM CuCl_2_ or 50 mM DTT_red_ were subjected as control (lanes 1, 2, 4 and 5). (B) Each pretreated AtPRK (4 μM) (oxidized, mono-oxidized, and reduced) was incubated with 8 μM AtCP12-2, 8 μM AtGapA and 0.5 mM NAD and subjected to gel filtration chromatography. (C) Peak I (9.2–10.4 mL), peak II (12.2–13.6 mL) and peak III (15.4–16.4 mL) fractions were subjected to SDS-PAGE. AtPRK (2 μg), AtGapA (2 μg) and AtCP12 (8 μg) were loaded as control. (D) Redox states of AtPRK in the peaks I and II fractions of the mono-oxidized sample were examined by nonreducing SDS-PAGE and immunoblotting. (E) Complex formation abilities of oxidized or reduced AtPRK Cys mutants (CCSS oxidized, CCSS reduced, SSCC oxidized, and SSCC reduced) were examined using gel filtration chromatography.

As described above, AtPRK activity was not significantly decreased when the C-terminal Cys pair was oxidized under the examined conditions (**Fig. 7A**, **B**). We then investigated the effects of Trx on the complex formation with *Arabidopsis* CP12-2 (AtCP12) and *Arabidopsis* GAPDH A subunit (AtGapA) to gain further insights into the regulatory mechanism of PRK in plant chloroplasts. AtPRK was prepared at different redox states, and they were mixed with oxidized AtCP12, AtGapA and NAD^+^. The redox states of pretreated AtPRKs and AtCP12 were examined using SDS-PAGE (**Fig. 9A**). After incubation, the protein mixture was subjected to gel filtration chromatography. Although AtPRK treated with AtTrx-*f*1 and DTT_ox_ contained a mixture of all three states (**Fig. 9A**, lane 7), it was used as the sample representing mono-oxidized AtPRK to simplify the data interpretation because other two AtPRK samples did not contain this form.

Three specific peaks were observed in the chromatogram (**Fig. 9B**), and their composition was analyzed by SDS-PAGE. Peak II was composed of AtPRK, AtGapA and CP12, irrespective of the redox states of AtPRK (**Fig. 9C**, lanes 5, 8 and 11). When AtPRK was in the mono-oxidized form, peak I also contained these three proteins. The estimated molecular weights of peaks I, II and III were 441,000, 110,000 and 29,600, respectively, as assessed by calibration with protein standards (**Fig. 9B**, **Supplementary Fig. S3**). Based on the calculated (482,000) and estimated (around 500,000; Marri et al. 2008, 2009) molecular weights of the complex and SDS-PAGE profile shown in **Fig. 9C**, we concluded that peak I corresponds to the GapA/CP12/PRK complex. When AtPRK was in the reduced form, the complex at peak I was not detected (**Fig. 9B**, black trace). Although the complex was also observed with the oxidized form of AtPRK, the height of peak I was very low compared with the sample containing the mono-oxidized AtPRK (**Fig. 9B**, blue and red traces).

We next checked the redox states of AtPRK in the peaks I and II fractions of the mono-oxidized sample. Although the calculated molecular weight of the AtPRK dimer was 79,000, a previous study showed that the oxidized AtPRK dimer was eluted in the mass region of 110 kDa during the gel filtration chromatography (Marri et al. 2005). Therefore, a free AtPRK dimer must be included in the peak II fraction. AtPRK was present as both the oxidized and mono-oxidized forms in the peak II fraction, but it was mostly present as the mono-oxidized form in the peak I fraction (**Fig. 9D**). These results indicate that mono-oxidized AtPRK has a high ability to form the complex. We also examined the ability to form the complex using AtPRK_CCSS_ and AtPRK_SSCC_ mutants and confirmed that only oxidized AtPRK_SSCC_ formed the complex efficiently (**Fig. 9E**, blue trace). This result strongly supports our conclusion. Taken together, Trx-mediated redox regulation of AtPRK is vital for complex formation/dissociation.

## Discussion

PRK is a redox-regulated enzyme in phototrophs. For this regulation, two Cys pairs, the N-terminal and the C-terminal pairs, which are conserved among class IA PRK, are thought to be critical (Hirasawa et al. 1998, Thieulin-Pardo et al. 2015). The N-terminal Cys pair located in the ATP-binding domain is believed to be regulated by Trx (Brandes et al. 1996). Previous studies suggested that the clamp loop region at the N-terminal side of the molecule is involved in disulfide bond formation between these Cys residues (**Supplementary Figs. S1, S2**; Gurrieri et al. 2019). However, PRK from cyanobacteria is also reported to be regulated by Trx (Hackenberg et al. 2018), although the clamp-loop region is not conserved among cyanobacteria (**Supplementary Figs. S1, S2**).

 It has already been shown that oxidants and reductants affect PRK activity or the formation of the supramolecular complex with CP12 and GAPDH (Wolosiuk and Buchanan 1978, Hirasawa et al. 1998, Wedel and Soll 1998, Geck and Hartman 2000, Kobayashi et al. 2003, Marri et al. 2005, 2009, 2014, Gurrieri et al. 2019, McFarlane et al. 2019). However, there has been no report so far that focused on the redox states of both the N-terminal and the C-terminal Cys pairs of the PRK molecule.

In this study, we showed that Trx alters the redox states of the C-terminal Cys pair of both A.7120_PRK and AtPRK (**Figs. 1**, **2**, **6**). Although the C-terminal Cys pair is located far from the active site and the oxidation of the C-terminal Cys pair did not significantly affect the activity of AtPRK, the activity of A.7120_PRK was partially suppressed (**Figs. 4**, **7**). In cyanobacterial PRK, oxidation of the C-terminal Cys pair might change the mobility of the adjacent helix and prevent crosstalk between helices and the active site (Wilson et al. 2019). Although Trx did not significantly affect the activity of AtPRK in this study, Trx-dependent activation of AtPRK and PRK from *Chlamydomonas reinhardtii* (Marri et al. 2009, Gurrieri et al. 2019) was previously reported. In these reports, the redox states of Cys pairs in PRK molecules were unclear. Trx, therefore, might regulate PRK activity under a particular condition in these organisms. Because fully oxidized PRK showed no activity (**Figs. 4**, **7**), the binding of ATP to the P-loop at the catalytic site may be inhibited by disulfide bond formation at the N-terminal Cys pairs.

 In *Arabidopsis*, the light-dependent change of the redox state of PRK has been reported (Perez-Ruiz et al. 2017). However, this study did not identify the Cys pair associated with the redox-dependent change in the enzyme molecule. Our in vivo studies indicate that PRK in cyanobacteria and plants takes the mono-oxidized form under dark conditions and shifts to the reduced form in the light (**Figs. 5**, **8**). Because the C-terminal Cys residues were easily reduced/oxidized by Trx (**Figs. 2**, **6**), they must be responsible for the redox state change of the enzyme molecule in response to light in vivo. A previous report that one of the N-terminal Cys residues was at the reduced form in the darkened leaves of *Pisum sativum* supports this idea (Howard et al. 2008). When only the C-terminal Cys pair formed the disulfide bond, this mono-oxidized form of PRK still showed activity (**Figs. 4**, **7**). This might be the reason why CP12 is essential for the complete deactivation of PRK under dark conditions. In cyanobacteria, both the reduced and the mono-oxidized forms were observed under light conditions (**Fig. 5A**, lanes 4 and 5), implying that Trx finely regulates PRK activity in response to light intensity.

 PRK is deactivated by forming a complex with CP12 and GAPDH under dark conditions (Howard et al. 2008). In addition, this complex formation is considered relevant for the protection of PRK from protein degradation (Elena Lopez-Calcagno et al. 2017). Our results showed that disulfide bond formation at the C-terminal Cys pair, but not the N-terminal Cys pair, is required to form the supramolecular complex with CP12 and GAPDH (**Fig. 9B**, **D**, **E**). These results are consistent with previous reports (Thieulin-Pardo et al. 2015, Yu et al. 2020). PRK binds to the N-terminal region of the oxidized CP12 as a dimeric form. In the three-dimensional structure of AtPRK dimer (PDB code 6H7H), the N-terminal Cys pairs were reduced in both subunits, but a C-terminal Cys pair forms a disulfide bond in one subunit (**Supplementary Fig. S1**). Although the overall structure and the position of Arg residues that interact with acidic residues from CP12 are highly identical, there are differences in the side-chain orientation of the amino acids in the loop containing the C-terminal Cys pair (**Supplementary Fig. S4**). A previous study has shown that the amino acid residues in this loop interact with that of the opposing subunit (Wilson et al. 2019). In addition, the conformation of this loop is considered to be stabilized by the disulfide bond formed at the C-terminal Cys pair (Wilson et al. 2019). A decrease in flexibility of PRK dimer might assist complex formation with CP12 and GAPDH.

The N-terminal and C-terminal Cys residues are widely conserved among class IA PRK (**Supplementary Fig. S2**). In contrast, the N-terminal Cys pair is well conserved, but the C-terminal Cys pair is not among class IB PRK found in chromista, dinoflagellates and haptophytes (**Supplementary Fig. S2**). Interestingly, the GAPDH/CP12/PRK complex has not been reported in these organisms; moreover, some of these organisms do not have CP12 gene (Jensen et al. 2017, Wilson et al. 2019). These facts also support the idea that the C-terminal Cys pair of PRK is involved in the interaction with CP12. PRK from land plants, green algae, β-cyanobacteria and red algae belong to class IA (Wilson et al. 2019). While the length between the N-terminal Cys pair is different among these organisms, the region around the C-terminal Cys pair is well conserved (**Supplementary Fig. S2**). Moreover, the red algae *Cyanidioschyzon merolae* lacks one Cys residue at the N-terminus (**Supplementary Fig. S2**). Therefore, Trx may also regulate the C-terminal Cys pair in these organisms.

In this study, we have shown that Trx regulates the C-terminal Cys pair of PRK in cyanobacteria and plants. In cyanobacteria, we also found that the redox state of the C-terminal Cys pair of PRK finely regulates the activity. Furthermore, our results showed that the redox regulation of PRK is required for complex formation with CP12 and GAPDH. Although it has been pointed out that the regulation of PRK activity is dependent upon Trx, our results show that Trx is important not only for the activity regulation of PRK but also for protein–protein interaction to form the GAPDH/CP12/PRK complex.

## Materials and Methods

### Expression and purification of recombinant proteins

DNA fragments encoding PRK from *A.*7120 (A.7120_PRK) *(alr4123)*, the mature protein region of PRK from *Arabidopsis* (AtPRK) *(At1g32060)* (corresponds to *Spinacia oleracea* PRK determined by Edman sequencing; Porter et al. 1986), the mature protein region of GapA (AtGapA) *(At3g26650)* and CP12-2 (AtCP12) *(At3g62410)* from *Arabidopsis* described in the previous report (Yu et al. 2020) were cloned into a pET-21a expression vector (Novagen). These plasmids were introduced into *Escherichia coli* strain BL21(DE3) for protein expression. Culture, induction of protein expression and protein purification were performed as described previously (Mihara et al. 2017, Yoshida and Hisabori 2017). In the case of AtGapA, protein expression was induced by 50 μM isopropyl β-d-thiogalactopyranoside. A.7120_PRK was purified using anion exchange chromatography and hydrophobic interaction chromatography. AtPRK, AtGapA and AtCP12 were purified using anion exchange chromatography, hydrophobic interaction chromatography and gel filtration chromatography. Their specific activities confirmed the correct status of PRK and GapA: A.7120_PRK, 222 ± 17 U/mg; AtPRK, 265 ± 24 U/mg; and AtGapA, 56.2 ± 2.3 U/mg, respectively, under their standard assay conditions. The recombinant proteins A.7120_Trx-*m*1 *(alr0052)* from *A.*7120, AtTrx-*f*1 *(At3g02730)* and AtTrx-*m*2 *(At4g03520)* from *Arabidopsis* were prepared as described previously (Yoshida et al. 2015, Mihara et al. 2017, Yoshida and Hisabori 2017). The protein concentrations were determined using BCA protein assays (Thermo Fisher Scientific, Waltham, MA, USA).

Cys substitutions with Ser in PRK were performed by introducing site-specific mutations into the DNA sequences using the PrimeSTAR Mutagenesis Basal Kit (Takara, Kusatsu, Japan). The primers used for site-directed mutagenesis are shown in **Supplementary Table S1**. These PRK mutants were expressed and purified similarly to the wild-type. For some Cys mutants, 25% saturated ammonium sulfate was used for hydrophobic interaction chromatography. These mutants were eluted using a 25–0% inverse gradient of ammonium sulfate in the buffer.

### Oxidation and reduction of PRK

PRK (2 µM) was incubated with 100 µM diamide, 50 µM CuCl_2_, 50 mM DTT_red_, 1 µM Trx and 10 mM DTT_ox_, or 1 µM Trx and 5–500 µM DTT_red_ in the buffer containing 50 mM Tris-HCl (pH 7.5) and 50 mM NaCl. A.7120_PRK or AtPRK were reacted at 30°C or 25°C, respectively. After incubation for 30 min, proteins were precipitated with 10% (w/v) trichloroacetic acid (TCA) and washed with ice-cold acetone. Precipitates were then suspended in SDS sample buffer containing 62.5 mM Tris-HCl (pH 6.8), 2% (w/v) SDS, 7.5% (v/v) glycerol and 0.01% (w/v) bromophenol blue and the thiol-modifying reagent. PEO (2 mM) or 2 mM AMS was used as a thiol-modifying reagent for A.7120_PRK or AtPRK, respectively. After modifying free thiols for 30 min at room temperature, proteins were separated by nonreducing SDS-PAGE using 10% (w/v) a polyacrylamide gel with 8 M urea in the separating gel. For Trx-dependent reduction assay of PRK, A.7120_PRK (40 µM) or AtPRK (25 µM) was treated with 100 µM diamide or 50 µM CuCl_2_, respectively, and desalted before reaction with Trxs.

### PRK and GAPDH activity assay

PRK activity assays were performed as described previously (Kobayashi et al. 2003) with slight modifications. PRK (2 µM) was incubated with 50 mM DTT_red_, 100 µM diamide, 50 µM CuCl_2_ or, 10 mM DTT_ox_ and 1 µM Trx for 30 min. After incubation at 30°C for A.7120_PRK or 25°C for AtPRK, 10 nM PRK was added to the reaction mixture containing 100 mM Tris-HCl (pH 7.5), 100 mM KCl, 10 mM MgCl_2_, 200 µM nicotinamide adenine dinucleotide, reduced (NADH), 0–2 mM ATP, 2.5 mM phospho (enol) pyruvate, 4.5–7 units/mL of lactate dehydrogenase, 3–5 units/mL of pyruvate kinase and 1 unit/mL of ribose 5-phosphate isomerase. The reaction was initiated by the addition of 2 mM ribose 5-phosphate, and the consumption of NADH was monitored at Abs340 at 30°C for A.7120_PRK or 25°C for AtPRK. The molar extinction coefficient for NADH of 6200 M^−1^ cm^−1^ was used to calculate the consumption of NADH. GAPDH activity assays were performed as described previously (Sparla et al. 2002).

### Identification of redox-regulated Cys residues by peptide mapping analysis

A.7120_PRK (2 µM) was treated with 10 mM DTT_ox_ and 1 µM A.7120_Trx-*m*1 in the buffer containing 50 mM Tris-HCl (pH 7.5) and 50 mM NaCl for 30 min at 30°C. The proteins were TCA-precipitated, labeled with 2 mM PEO and subjected to nonreducing SDS-PAGE. The gel band of the mono-oxidized PRK was excised and divided into two pieces. One gel slice was treated with 10 mM DTT_red_ before alkylation of free thiols with IAA. The details are mentioned in the figure legends. In-gel digestion and mass spectrometry analysis were performed as previously described (Yoshida et al. 2015). Results were queried with the Mascot search engine (Matrix Science, Boston, USA) to identify matched peptides. The parameters were as follows: Database, NCBIprot 20191122; enzyme, trypsin; taxonomy, other bacteria; fixed modifications, carbamidomethyl (C); variable modifications, oxidation (M); mass values, MH+ and monoisotopic; peptide mass tolerance, 50 ppm; max missed cleavages, 1.

### Bacterial strains and growth conditions

*A.*7120 cells were grown at 30°C in BG11 medium (Rippka et al. 1979) supplemented with 20 mM HEPES-NaOH (pH 7.5) under continuous light (30 µmol photons m^−2^ s^−1^ for low light or 200 µmol photons m^−2^ s^−1^ for high light) conditions. Liquid cultures were bubbled with air containing 1% (v/v) CO_2_. *trxM1* knockout mutant was constructed as described previously (Mihara et al. 2018).

### Determination of in vivo redox states of cyanobacterial PRK

Three-day cultures under low light or 2-d cultures under high light of WT cells were precipitated with 10% (w/v) TCA and washed with ice-cold acetone. For dark conditions, the culture tube was covered with aluminum foil for 3 h before TCA precipitation. The cells were treated with 25 mM DTT_red_ for 10 min at room temperature before TCA precipitation for the DTT treatment condition. Precipitates were then suspended in SDS sample buffer containing 4 mM PEO. After labeling for 40 min at room temperature, protein samples were boiled at 95°C for 5 min and centrifuged at 20,400×*g* for 10 min. Proteins in the supernatants were then separated by nonreducing SDS-PAGE using 10% (w/v) polyacrylamide gel with 8 M urea in the separating gel and blotted onto polyvinylidene difluoride membranes. Immunoblotting was performed using PRK polyclonal antibodies against recombinant AtPRK. The chemiluminescence of the horseradish peroxidase-conjugated secondary antibody was detected using an LAS 3000 instrument (Fujifilm, Tokyo, Japan).

### Determination of in vivo redox states of plant PRK

*Arabidopsis thaliana* wild-type plants (Col-0) were grown in soil in a controlled growth chamber (light intensity, 70–80 µmol photons m^−2^ s^−1^; temperature, 22°C; relative humidity, 60%; 16 h day/8 h night) for 4 weeks. For determining PRK redox states under dark and light conditions, plants were dark-adapted for 8 h or irradiated at 700 µmol photons m^−2^ s^−1^ for 15 min. The sample preparation was performed according to Yoshida and Hisabori (2019). PRK was detected by nonreducing SDS-PAGE and immunoblotting, as shown above.

### GapA/CP12/PRK complex formation

AtCP12 (20 µM) was treated with 50 µM CuCl_2_ at 25°C for 10 min. AtPRK_WT_ (20 µM), AtPRK_CCSS_ (20 µM) or AtPRK_SSCC_ (20 µM) was treated with 50 mM DTT_red_ or 50 µM CuCl_2_ at 25°C for 10 min. After the above treatments, oxidants and reductants were removed using PD MidiTrap G-25. AtPRK_WT_ (20 µM) was incubated with 5 µM AtTrx-*f*1 and 30 mM DTT_ox_ at 25°C for 1 h to prepare mono-oxidized PRK. AtTrx-*f*1 and DTT_ox_ were removed by gel filtration chromatography using a Superdex 200 10/300 GL column (GE Healthcare, Chicago, USA). Each treated AtPRK (4 µM) was mixed and incubated with oxidized AtCP12 (8 µM), 8 µM AtGapA and 0.5 mM NAD at 25°C for 3 h in the buffer containing 50 mM Tris-HCl (pH 7.5) and 50 mM NaCl. The complex formation was checked using gel filtration chromatography. Gel filtration chromatography and calibration of a Superdex 200 10/300 GL column were performed as described previously (Mihara et al. 2018).

## Supplementary Material

pcac050_Supp

## Data Availability

All data are incorporated into the article and its online supplementary material.
