# Supplementary material for: The Importance of the C-Terminal Cys Pair of Phosphoribulokinase in Phototrophs in Thioredoxin-Dependent Regulation
Source: Plant Cell Physiol. 2022 Apr 12;63(6):855–68. doi: 10.1093/pcp/pcac050 (PMC9199185; doi:10.1093/pcp/pcac050)
Supplement: pcac050_Supp [file pcac050_supp.zip › pcp-2022-e-00066-File011.pdf]

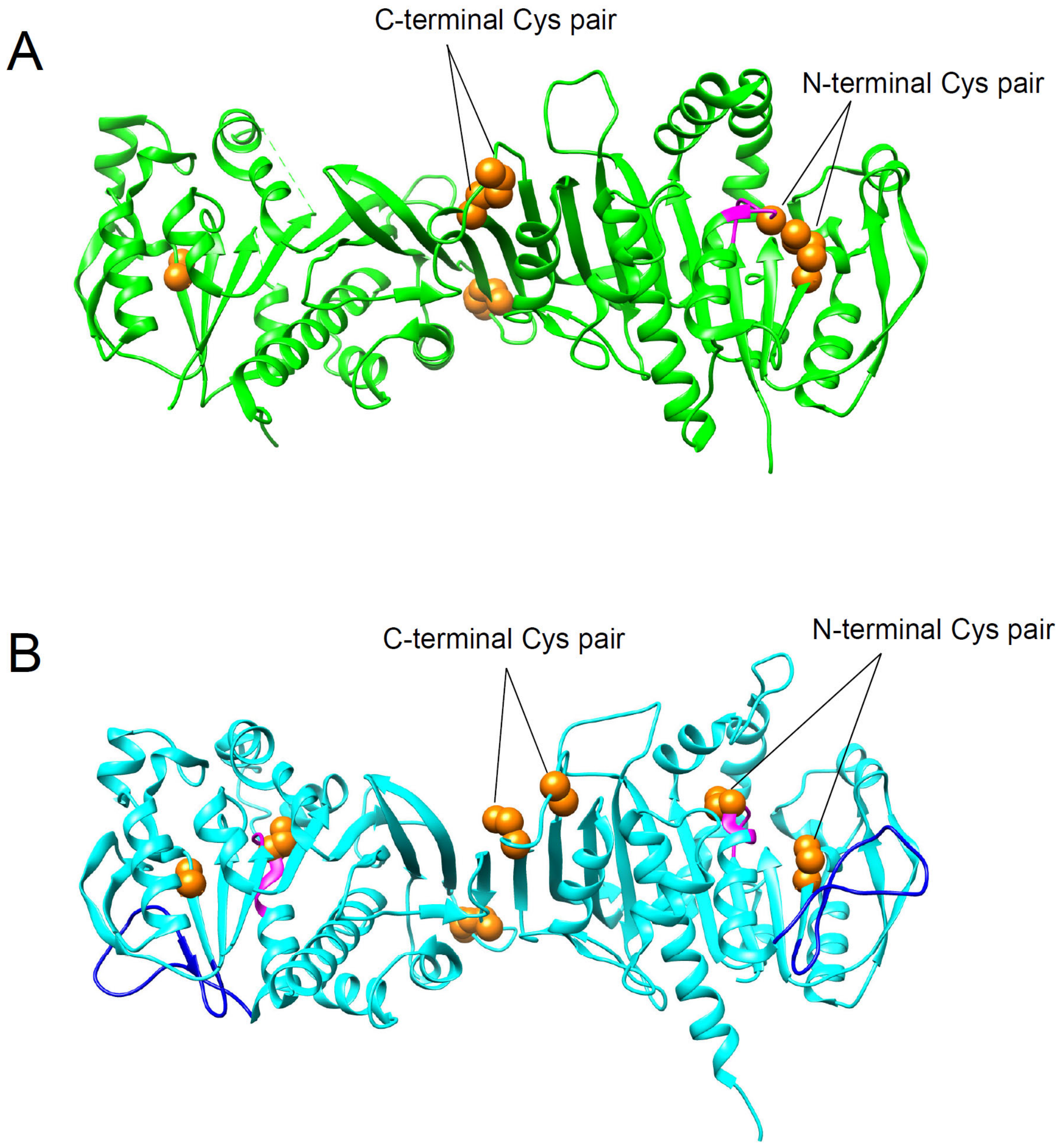

Fig. S1. Structure of cyanobacterial and plant PRK.

PRK from cyanobacteria *Synechococcus* sp. PCC 6301 (PDB code 6HZK) (*A*) and AtPRK (PDB code 6H7H) (*B*) are colored green and cyan, respectively. The clamp loop, p-loop, and Cys residues are highlighted in blue, magenta, and orange, respectively.

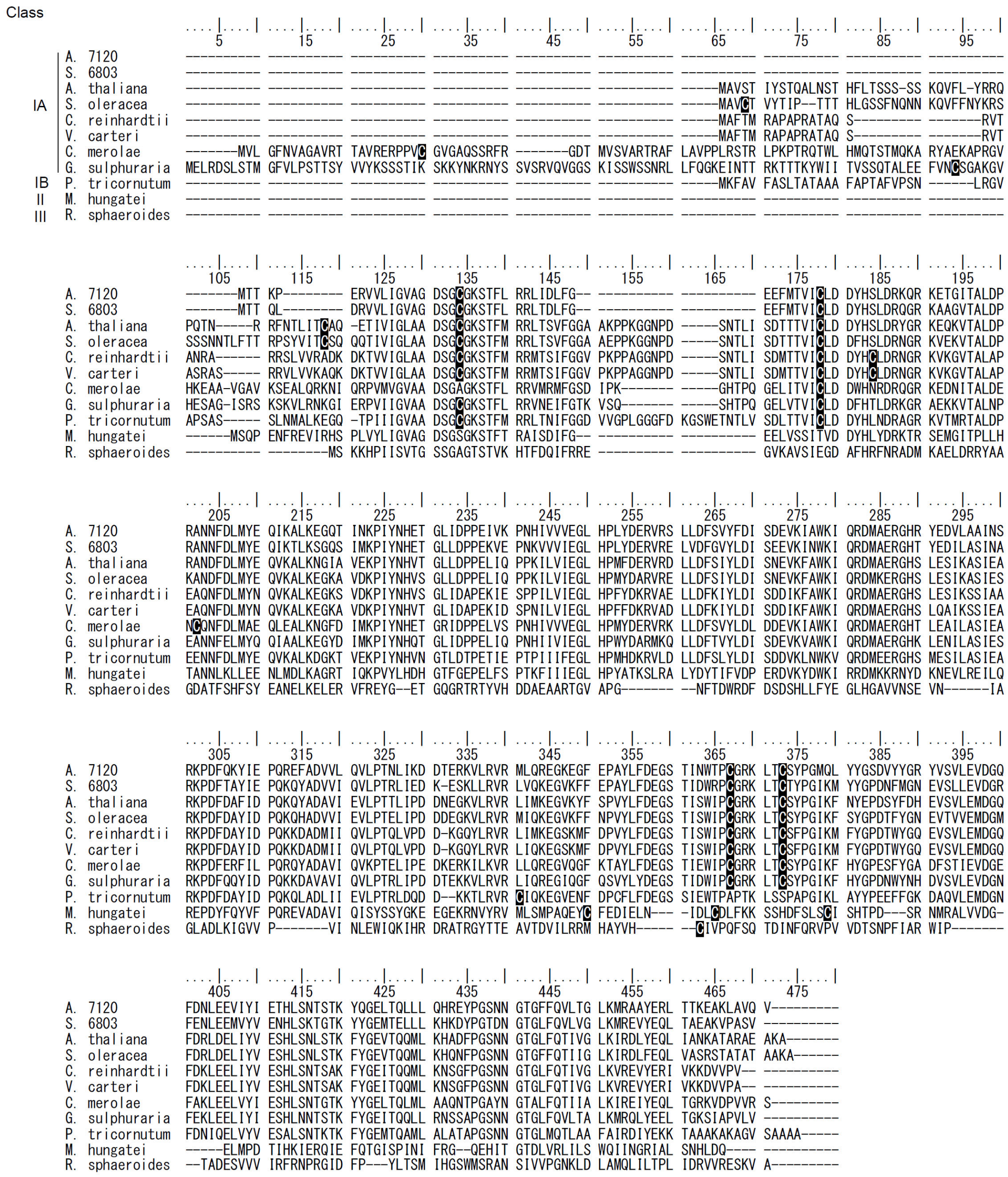

Fig. S2. Sequence alignment of PRK.

Multiple sequence alignment was performed using ClustalW. The accession numbers of proteins are all4123 (*A.*7120), sll1525 (*Synechocystis* sp. PCC 6803; *S.* 6803), At1g32060 (*Arabidopsis thaliana*), XP_021851648.1 (*Spinacia oleracea*), Cre12.g554800.t1.2 (*Chlamydomonas reinhardtii*), Vocar.0007s0361.1.p (*Volvox carteri*), XP_005535773.1 (*Cyanidioschyzon merolae*), XP_005707300.1 (*Galdieria sulphuraria*), XP_002186456.1 (*Phaeodactylum tricornutum*), WP_011447825.1 (*Methanospirillum hungatei*), and P12033.2 (*Rhodobacter sphaeroides*). Cys residues are highlighted in black.

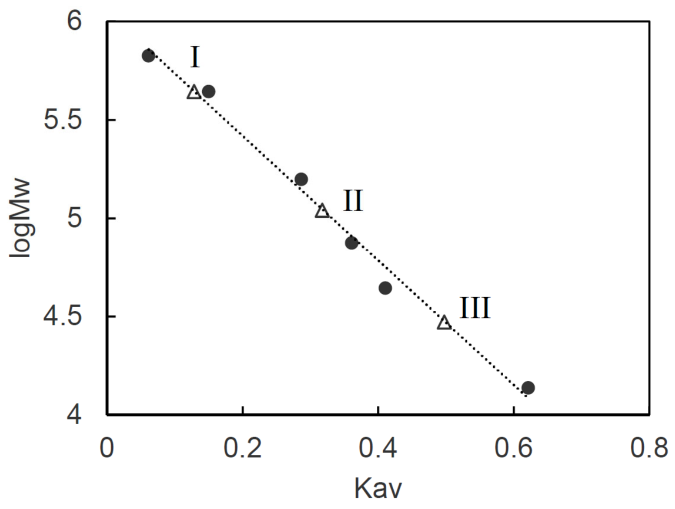

Fig. S3. Calibration of the gel filtration column and estimation of molecular mass.
Superdex 200 column was calibrated using thyroglobulin (669 kDa), ferritin (440 kDa), aldolase (158 kDa), conalbumin (75 kDa), ovalbumin (44 kDa), and ribonuclease A (13.7 kDa). The filled circles represent the above protein standards, and the open triangles represent peaks I, II, and III shown in Figure 8B.

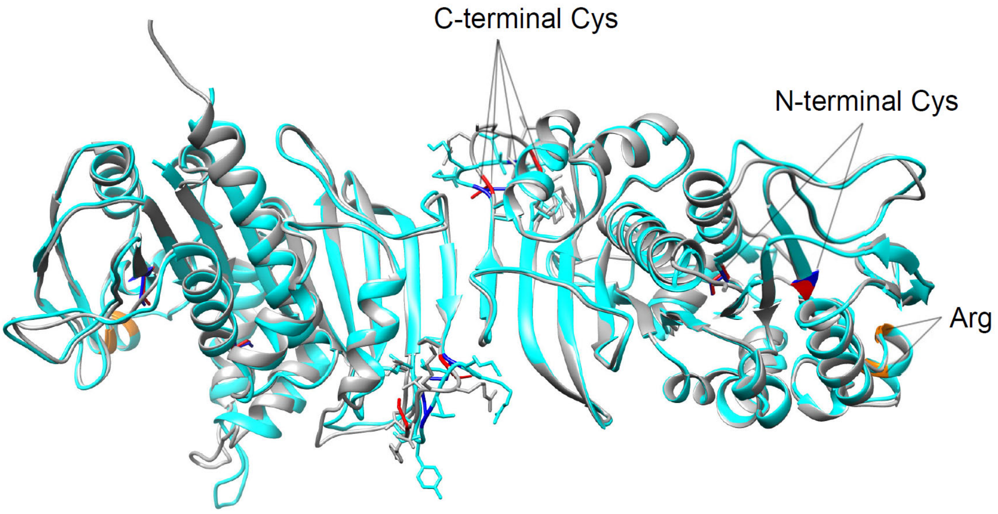

Fig. S4. Structural comparison of AtPRK subunits with and without a C-terminal disulfide bond. The structure of subunit A containing no disulfide bond (PDB code 6H7H, subunit A) and the subunit B containing a disulfide bond at the C-terminus (PDB code 6H7H, subunit B) were compared using UCSF Chimera software. The subunits A and B are colored gray and cyan, respectively. The Cys residues of subunits A and B are highlighted in red and blue, respectively. The Arg residues which forms hydrogen bonds with acidic amino acids from CP12 are highlighted in orange. The side chains of amino acids in the loop containing the C-terminal Cys residues are shown in stick representation.

Table S1. The primers used for the site-directed mutagenesis of PRK

A.7120_PRK C19S	Forward	5' TCCGGGTCCGGTAAATCTACGTTTTTG 3'
A.7120_PRK C19S	Reverse	5’ TTTACCGGACCCGGAGTCTCCGGCTAC 3’
A.7120_PRK C41S	Forward	5' GTCATCTCTTTAGATGACTACCATTCT 3’
A.7120_PRK C41S	Reverse	5' ATCTAAAGAGATGACCGTCATAAACTC 3’
A.7120_PRK C230S	Forward	5' ACTCCTTCCGGACGTAAGCTTACCTGT 3’
A.7120_PRK C230S	Reverse	5' ACGTCCGGAAGGAGTCCAGTTAATTGT 3’
A.7120_PRK C236S	Forward	5' CTTACCTGTTCTTACCCAGGAATGCAG 3’
A.7120_PRK C236S	Reverse	5' GTAAGAAGAGGTAAGCTTACGTCCGGA 3’
AtPRK C15S	Forward	5' TCTGGCTCCGGCAAAAGTACCTTTATG 3'
AtPRK C15S	Reverse	5' TTTGCCGGAGCCAGAGTCAGCAGCTAG 3'
AtPRK C54S	Forward	5' GTGATCTCTCTTGATGATTACCATTCT 3'
AtPRK C54S	Reverse	5' ATCAAGAGAGATCACAGTGGTCGTGTC 3'
AtPRK C243S	Forward	5' ATTCCTTCCGGCCGCAAACTCACTTGC 3'
AtPRK C243S	Reverse	5' GCGGCCGGAAGGAATCCACGAGATGGT 3'
AtPRK C249S	Forward	5' AAACTCACTTCCTCGTACCCTGGCATC 3'
AtPRK C249S	Reverse	5' CGAGGAAGTGAGTTTGCGGCCGGAAGG 3'
